# Supporting preschoolers’ cognitive development: Short‐ and mid‐term effects of fluid reasoning, visuospatial, and motor training

**DOI:** 10.1111/cdev.13642

**Published:** 2021-08-20

**Authors:** Valentina Gizzonio, Maria Chiara Bazzini, Cosima Marsella, Pamela Papangelo, Giacomo Rizzolatti, Maddalena Fabbri‐Destro

**Affiliations:** ^1^ Istituto di Neuroscienze Consiglio Nazionale delle Ricerche (CNR) Parma Italy

## Abstract

Cognitive abilities are essential to children's overall growth; thus, the implementation of early and effective training interventions is a major challenge for developmental psychologists and teachers. This study explores whether an intervention simultaneously operating on fluid reasoning (FR), visuospatial, narrative, and motor abilities could boost these competencies in a group of Italian preschoolers (*N* = 108, 54 males 54 females, Age_mean_ = 4.04). FR and visuospatial abilities showed training‐related increases at the end of the training and 1‐year follow‐up (moderate effect size). Interestingly, positive correlations with working memory and mathematical abilities were found. Beyond their scientific relevance, the short‐ and long‐term effects provide fundamental indications for designing and implementing educational programs dedicated to preschoolers.

**Abbreviations**
CI: comprehension of instruction
FR: fluid reasoning
I‐BST: Bus Story Test
IQ: intelligence quotient
IS: Information Scores
MD: Memory for Designs
PH: phonological processing
PS: processing speed
SC: Subordinate Clauses
SL: Sentence Length
SN: speeded naming
SR: Sentence Repetition

The preschool period (3–6 years) is a time of rapid growth along which many changes happen in children's development. During this period, children learn new skills belonging to fundamental domains like social and emotional abilities and cognitive development (Haddad et al., 2019). Cognitive development refers to the process of growth and change in intellectual/mental abilities such as thinking, reasoning, and understanding, including the acquisition and consolidation of knowledge. Children this age begin to learn questioning, spatial relationships, problem‐solving, imitation, number sense, and symbolic play. Such a constellation of functions is vital to the child's overall growth and development (Rueda et al., 2005; Thorell et al., 2009; Wass et al., 2011). Thus, early and effective training interventions—possibly embeddable in everyday life—are among the major challenges for developmental psychologists and teachers (see Goswami, 2015; Kuhn & Siegler, 1998).

A broad range of cognitive competencies progresses during early childhood. Among them, we focused our attention on fluid reasoning (FR), visuospatial, linguistic, and motor abilities, intending to propose to preschool children an intervention simultaneously touching upon all these competencies.

Problem solving is the cognitive process for achieving a goal when a solution method is not obvious to the problem solver (Lovett, 2002; Mayer, 1992). It is part of the more general domain called fluid intelligence or FR. According to the Cattell–Horn–Carroll theory, FR is defined as the deliberate but flexible control of attention to solve novel *on‐the‐spot* problems that cannot be performed by relying exclusively on habits, previously learned schemas, and scripts (see Schneider & McGrew, 2018). It is an essential component of cognitive development (Goswami, 1992) since this capacity serves as a scaffold for children, helping them acquire other abilities (Blair, 2006; Cattell, 1971, 1987; Ferrer et al., 2009).

FR predicts performance on a wide range of cognitive activities, including performance in school, university, and cognitively demanding occupations (Gottfredson, 1997), and some studies have demonstrated that low FR in children is a predictor of academic difficulties (Lynn et al., 2007; Nisbett, 2009).

Whether FR can be improved with training has been investigated with different strategies across the lifespan (Plemons et al., 1978). While studies on healthy adults yielded disappointing results (e.g., Detterman & Sternberg, 1982), FR has been successfully trained in children (Christoforides et al., 2016; Hamers et al., 1998; Hernstein et al., 1986; Klauer et al., 2002; Niklas et al., 2016), promoting early math skill development in kindergarten and elementary school age. In particular, Bergman Nutley et al. (2011) administered to 4‐year‐old children computerized training of either non‐verbal reasoning, working memory, a combination of both, or a placebo version of the combined training. Only the non‐verbal reasoning training significantly impacts FR, while smaller gains on problem‐solving tests were seen in the other groups. Similarly, Mackey et al. (2011) compared in children (aged 7–9) the effects of two computerized training programs focused on FR and processing speed (PS). Both training programs led to significant improvements in the cognitive domain targeted explicitly by the training, with no cross‐talk between FR and PS. Overall, evidence was provided about the possibility of improving FR (see for a review Buschkuehl & Jaeggi, 2010) in children (Jaeggi et al., 2008, 2011; Sternberg, 2008), adults (Ball et al., 2002), and atypically developing populations (Klingberg et al., 2002, 2005). However, no indication has been provided to date how long these training effects last (Jaeggi et al., 2008; Spitz, 1992). Moreover, computerized training is performed individually by children (see Mackey et al., 2011), lacking the motivational and imitative drives typical of the social environment in which children learn together with their peers.

Strictly related to FR, spatial skills are another critical component of cognitive development in children. They are considered an umbrella term for a constellation of inter‐related abilities as, for example, the capacity to mentally manipulate the object information or visualize how objects fit together (see Uttal et al., 2013). Better performers in spatial tasks typically have higher mathematical abilities, as proved by behavioral and neuropsychological measures (Guay & McDaniel, 1977; Mix & Cheng, 2012; Xie et al., 2020).

The effectiveness of training interventions on spatial skills has been previously shown (Kim et al., 2018; Verdine et al., 2014). The time spent playing with assembly toys has a pivotal role (Jirout & Newcombe, 2015). Interestingly, contextual elements emerged as relevant for the training outcome beyond the spatial content of these activities. Casey et al. (2008) combined a block‐building intervention with storytelling procedures, demonstrating that storytelling enhances spatial learning. Grounding spatial tasks in a storytelling context could produce greater retention and recall of the information (Bower & Clark, 1969) and engage children's motivation in solving the spatial task (Casey et al., 2008).

When exposed to narration, children experience a sort of continuum ranging from the discursive exposition in storytelling to their enactment in a play‐like situation (Nicolopoulou & Richner, 2007; Nicolopoulou et al., 2014). A series of research demonstrated that children's acquisition of oral language skills in their preschool years, including narrative ones, is a foundation for academic abilities such as reading comprehension, writing reports, and formulating oral presentations (Griffin et al., 2004; Kendeou et al., 2009; Lynch et al., 2008; Nicolopoulou et al., 2015; Reese et al., 2010). Thus, training narrative abilities at an early stage would help individuals to exploit at best later their language skills.

Even if out of the traditional cognitive domains, decades of psychological theory and research have established that motor abilities are strictly intertwined with cognitive development in infancy and early childhood (e.g., Adolph, 2008; Davis et al., 2011; Piaget, 1952). Since the earliest developmental stages, the unfolding of cognitive abilities appears influenced by the onset of corresponding motor skills (e.g., exploration capacity vs. locomotion, Lehnung et al., 2003). This link, however, is not limited to the early timing, as the two domains follow a comparable and progressive timetable (Bushnell & Boudreau, 1993) also during later development. Positive relations between motor and cognitive domains have also been supported by neuropsychological and neuroimaging studies (Diamond, 2000; Wassenberg et al., 2005).

The pathological counterpart of this interplay is represented by the cognitive impairments following a delay or deviance in targeting motor developmental milestones. For example, idiopathic toe walking is considered a precursor of developmental language and learning problems (Sala et al., 1999). Impaired motor function is a precursor of language acquisition problems and later attention skills (Amiel‐Tison et al., 1996; Hamilton, 2002).

While attempts to train the abovementioned abilities have been carried out mainly in isolation, that is, addressing one specific domain at a time, we sought to design an intervention touching upon all these domains. We enrolled 157 preschoolers (3–5 years old) and administered them a training procedure stimulating FR, visuospatial and motor skills, and narrative abilities. Children were subdivided into three groups, differing for the activities they were exposed to during training. A neuropsychological battery was administered before and after the training and at 1‐year follow‐up to evaluate short‐term impacts and maintenance over time. While the first analysis can be considered confirmatory, as an immediate impact of the training is lagerly expected on some domains, the latter can be regarded as more exploratory, because it is far from granted that the training effects can resist after 1 year. Results will be discussed in light of the potential of preschool daily practice to potentiate emerging skills and prompt the acquisition of new ones fundamental for children's future learning and discoveries.

## METHODS

### Participants

In 2016, an initial sample of 157 preschoolers was enrolled in the study across five different kindergartens in Parma (Italy). Kindergartens in Italy are a preschool service for children from 3 to 5 years old, preceding the access to the primary school that happens at 6 years old. Informed written consent was obtained from the parents and oral consent from the children. The Local Ethical Committee approved this study (prot. n. 45017, 14‐12‐2015), which was conducted according to the Helsinki Declaration.

### Study design

The study was articulated in five different moments, including (1) an initial screening conducted on 157 children; (2) a neuropsychological evaluation administered before the intervention (T0); (3) the administration of a training (32 sessions), namely intervention; (4) a neuropsychological evaluation administered after the intervention, about 1 year after T0 (T1); and finally, (5) a follow‐up neuropsychological evaluation 1 year after T1 (T2). At T2, we administered additional tests to investigate the verbal and visuospatial working memory and the basic mathematical skills. The whole timeline lasted from 2016 to 2019.

Such experimental design aimed to obtain a global picture of children's skills before the treatment, after it, and 1 year later to identify the impact of the intervention on different domains, its maintenance over time, and the possible generalization to other domains not explicitly trained.

### Screening

All children were admitted to a screening evaluating cognitive and linguistic abilities to exclude individuals with intellectual disabilities or language difficulties, potentially compromising the reliability of the study results.

The intelligence quotient (IQ) was evaluated by the Leiter International Performance Scale‐Revised (Leiter‐R; Roid & Miller, 2002). The linguistic domain was investigated administering three subtests of the NEPSY‐II (Korkman et al., 2011): comprehension of instruction (CI), phonological processing (PH), and speeded naming (SN).

Following the screening assessment and teachers’ reports, 13 children were not included in the training. Three had started a clinical evaluation at the local Neuropsychiatry Service, two were bilingual with difficulties in Italian language comprehension and production, and one was certified for visual and auditory difficulties. The remaining seven children could not adhere to the screening procedure and completed only partially the required tests preventing us from their inclusion in the experimental sample. A minimum threshold of 70 was required for Leiter‐R, and a score greater than five was needed for any of the linguistic subtests (CI, PH, SN). However, none of the children had scores below these thresholds. At the end of the screening, the sample to‐be‐included in the study was 144 children (78 girls; 66 boys) with ages from 3 to 5 years (*M* = 4 year1 month, *SD* = 6 month).

### Group assignment

After the screening, participants were subdivided into three groups according to the type of toys used during the training. Children playing with modular toys were required to assemble different pieces and were included in the Assembling group (*A*); children receiving plush toys were assigned to the Plush group (*P*); remaining children composed the Control group (*Ctrl*). While the first two groups would have been later administered with specific training, children of the control group continued curricular programs without attending any extra activity. The inclusion of a training‐free group let us control for the spontaneous development of cognitive abilities over time in a sample of participants attending the same schools and curricular activities.

Since the intervention was distributed across five different schools, their heterogeneity (e.g., districts, teachers, class size) could introduce several potentially confounding factors in our study. To account for most of them, we decided to balance the group numerosity within each school. Starting from this constraint, we first split the group according to age: children attending the first year of kindergarten and those attending the second year. Within each of these groups, we sorted children according to their IQ and then subdivided them into triplets. For each triplet (child 1, child 2, child 3), a computer‐generated sequence randomly assigned the three children to groups (e.g., PCA implies child 1 to P, child 2 to Ctrl, child 3 to A group).

This way, we warranted that groups were balanced in terms of IQ, and at the same time, they equally included the 2 years of kindergarten attendance, thus likely reflecting a further balance in terms of age. Since this procedure was replicated for each school, the overall sample benefited from the same balancing properties.

### Intervention

The intervention was conducted during the regular kindergarten hours. As school numerosity was quite different (range 18–51), we further subdivided the experimental groups (Assembling and Plush) into smaller groups of 6–9 children to balance among schools the potential effect of team working. One of the three developmental psychologists (MCB, PP, CM) conducting the intervention was randomly assigned to each group.

The intervention sessions (approximately 50 min each) took place in a dedicated room within the school twice a week. The 32 sessions composing the training were distributed over about 5 months. Children assigned to the control group, on the contrary, continued curricular programs without attending any extra activity.

Each experimental session was characterized by four moments: toy delivery, the introduction of the story by the experimenter, turn‐based interplay, quizzing children with questions whose answer requires the solution of logical tasks, and retelling.

**FIGURE 1:**
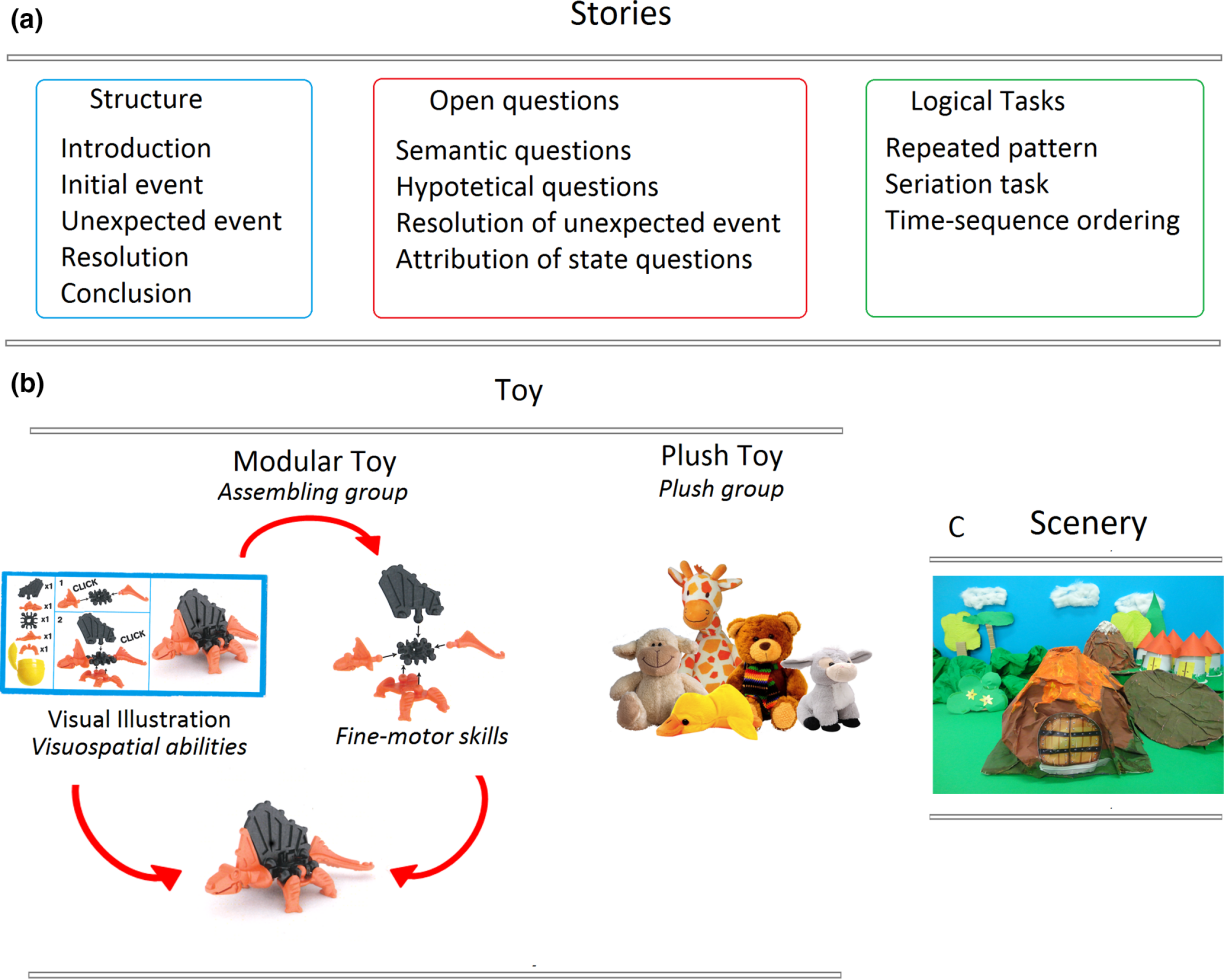
(a) Structure of the stories used during the training sessions. Each story contained open questions and logical tasks to stimulate the narrative domain and the emergence of problem‐solving capabilities. (b) Toys utilized during the training. Children of the Plush group received plush toys, while children of the Assembling group received a modular toy to be built following visual instructions. The Plush group's toy represented the same characters used by the Assembling group but in the "soft and big" version. (c) Scenery: a 3D set design was created for each story to provide a concrete context to the narration in which every child could act the story through his/her character

1) Toy delivery: At the beginning of each session, the experimenter gave children toys acting as characters of the story. Children of the Assembling group received a kit with pieces to construct a little toy and the visual instructions to be followed for its assembling (see Figure 1b). In the first 5 weeks, toys presented an increasing difficulty (from 2 to 7 pieces). Subsequently, the difficulty level of toys was randomized. On the other side, the Plush group received a stuffed toy bigger than those used by the first group and representing the same characters (Figure 1b). The choice of these two types of toys was intended at potentiating, within a common framework, the motor skills and visuospatial abilities in the Assembling group.
2) Stories: A set of 32 stories was used during the training. These were created by our team of psychologists maintaining the structural regularity of the narrative text (Levorato, 1988): introduction of the context and characters, an initial event that triggers the actions of the characters, several attempts to solve the problem, the consequences of such attempts, and finally a conclusion (see Figure 1a). The characters of each story corresponded to the toys delivered to children to make them actively participate in the story. An exemplar story used during the training can be found in Supporting Information. Scenery: A home‐made scenery was created for each story to provide children with a concrete space (e.g., a laboratory, the sea, or a forest) in which each child, through his character, could live and act the story Figure 1c shows an example of a 3D set used during the sessions.

3) Quizzing and interplay: Every story contained *questions* and *logical tasks* to stimulate the narrative domain and problem‐solving capabilities (see Figure 1a). In particular, four types of open questions were used: *semantic* (e.g., what is a scientist?), *hypothetical* (e.g., what will it happen then?), *resolution of unexpected events* (e.g., how can they cross the river?), and *attribution of state questions* (e.g., what does he/she think? what emotion does he/she feel?). Children were instructed to answer individually without reference to previous answers by other peers, thus promoting original responses. The experimenter repeated each answer enriching it with additional elements so to stimulate narrative competencies. While the open questions were answered individually, children solved the logical tasks collectively. These were subdivided into *repeated patterns, seriation, and time‐sequence ordering* tasks. *Repeated pattern* tasks required the child to understand a logical sequence and complete it considering the initially provided model. The *seriation* tasks required the child to compare elements (e.g., size, quantity, color) and identify the relations between them, recognizing the correct order. Finally, the *time‐sequence ordering task* required the child to reconstruct the temporal sequence respecting the logical and sequential order of events.

4) Retelling: Finally, children were invited to retell the story. In this schema, the toy becomes the physical bridge that lets children play the story. Indeed, they become part of the story *via* their characters, interact with other mates and characters, and succeed or fail collectively. This cooperative, active, and interactive scenario rendered the training more similar to a play context than a traditional trainer‐trainee relationship.

### Neuropsychological evaluation

We administered a neuropsychological battery at three time‐points, that is, before the treatment (T0), after it (T1), and at 1‐year follow‐up (T2).

During the training session, children received questions about the story and implying logical tasks to boost their problem solving. The underlying function, that is, FR, was evaluated by Raven's Colored Progressive Matrices (Raven, 1984). The test measures relational reasoning and is considered the most specifically designed test to measure fluid intelligence (Cotton et al., 2005).

The visuospatial and motor abilities were evaluated by administering two subtests of the NEPSY–II (Korkman et al., 2011). The *Block Construction subtest* provides a measure of the ability to mentally organize visual information by analyzing part‐whole relation when the information was presented spatially. In the *Imitating Hand Position subtest*, the child imitates various hand positions demonstrated by the examiner, thus obtaining an index of his/her performance in terms of visuomotor transformation.

Finally, children's answers were repeated and enlarged by the experimenter so to promote their storytelling. The linguistic/narrative competence was evaluated using *Information Scores* (*IS*), *Sentence Length* (*SL*), and *Subordinate Clauses* (*SC*) scores from the Bus Story Test (I‐BST; Renfrew, 1997). The IS measures how many information units of the original story the child uses during the retelling. The SL indexes the morpho‐syntactic complexity of the retelling, and the SC score depends on the number of utterances containing a subordinate clause.

An additional battery was administered only at T2, comprising working memory and mathematical abilities assessment. Visuospatial and verbal working memory abilities were evaluated by administering the *Memory for Designs* (MD) and *Sentence Repetition* (SR) subtests of NEPSY‐II (Korkman et al., 2011), respectively. Mathematical abilities were assessed using the TEDI‐MATH test (Van Nieuwenhoven et al., 2015). Table 1 summarizes the investigated competencies, the related tests and subtests, and the time‐points at which each test was administered.

**TABLE 1:** Neuropsychological battery. Investigated competencies, tests, subtests, and the timing (T0, T1, T2) of administration are reported. Visuospatial and Verbal Working Memory (WM) and mathematical skills are tested only at T2

Investigated competences	Tests and subtests	Time‐points
Fluid reasoning	Raven's Colored Progressive Matrices (RCPM)	T0, T1, T2
Visuospatial abilities	NEPSY‐II, Block Construction (VS)	T0, T1, T2
Fine motor abilities	NEPSY‐II, Imitating Hand Positions (FM)	T0, T1, T2
Linguistic/narrative competence	I‐BST, Information Scores (IS)	T0, T1, T2
	I‐BST, Sentence Length (SL)	T0, T1, T2
	I‐BST, Subordinate Clauses (SC)	T0, T1, T2
Visuospatial WM	NEPSY‐II, Memory for Designs (MD)	T2
Verbal WM	NEPSY‐II, Sentence Repetition (SR)	T2
Basic mathematical skills	TEDI‐MATH	T2

### Drop‐out

The children initially enrolled in the study were required to be 3 or 4 years old at T0, as they were attending the first or the second year of kindergarten at that time. At T1, they were still attending the same school, and all of them were recruited for re‐testing (144 T0, 144 T1). However, between T1 and T2, 36 children dropped out, including those who moved to different institutes or towns and those whose parents did not sign the informed consent for the follow‐up procedures. These 36 participants were excluded from the final sample to guarantee a complete dataset comprising pre and post‐intervention and follow‐up observations for all participants. Of the remaining 108 children, 41 remained for the whole study duration in kindergarten, while 67 moved to primary school.

### Data analysis and statistics

The final sample of children admitted to data analysis comprised those having complete evaluation across T0, T1, and T2 and was composed of 108 children. A factorial analysis was conducted on scores reported in Table 2, intended at verifying the homogeneity among groups at baseline in terms of age, initial cognitive, and linguistic levels. Gender balance was assessed as well *via* a chi‐squared test.

**TABLE 2:** Means and standard deviations of the measures collected during the screening

Screening evaluation	Assembling		Plush		Control	
	*M*	*SD*	*M*	*SD*	*M*	*SD*
Leiter‐R	127.4	13.7	122.9	11.9	122.3	13.7
Comprehension of instructions	9.6	2.6	9.4	2.9	9.8	2.8
Speeded naming	12.0	2.1	12.0	0.9	11.8	1.1
Phonological processing	10.7	2.4	10.3	3.0	10.2	2.6

Concerning the tests listed in Table 1, we admitted to the analysis the raw scores and not the ones normalized per age. This choice was driven by a limitation intrinsic to our experimental design. Indeed, most of the tests would require a normalization procedure based on the child's chronological age, with steps 365 days long. However, because 394 (±27) days interspersed on average between T0 and T1, the impact of age‐normalization would have been tremendously different across children, with some of them remaining in the same year of normalization, and others advancing of two (and not just one) years of normalization. We thus opted to consider raw values to overcome this paradox, being aware that raw values are supposed to increase over time due to the spontaneous development of children's abilities, even regardless of our intervention. However, we aimed at revealing that such an increase had been higher in the case of children belonging to the experimental groups.

For this reason, we did not consider in the analysis the absolute values recorded at T0, T1, and T2, but rather the relative increases observed at T1 and T2 against T0 (i.e., Delta 1: T1–T0, Delta 2: T2–T0). Delta 1 was intended to index the immediate effectiveness of the intervention for each child in each domain. At the same time, Delta 2 served to evaluate whether these increases were possibly maintained at the 1‐year follow‐up, selectively across groups. We did not account for T2–T1 because such a difference would be devoid of any effect directly linked to the training.

Statistical analysis was conducted with a one‐way factorial design, including a between‐subjects factor (group: *Ctrl*, *A*, *P*). All variables underwent the Shapiro–Wilk's *W*‐test for verifying the assumption of normality. Screening variables underwent a one‐way ANOVA or Kruskal–Wallis analysis to assess the homogeneity of groups in terms of baseline characteristics. For Delta scores, statistical parametric analyses were performed via ANCOVA with Group as between‐subject factor and screening scores (Age, IQ, CI, SN, PH) as covariates. Newman–Keuls correction for multiple comparisons was applied. In the case of non‐parametric tests, Kruskal–Wallis and Mann–Whitney post hoc were used accordingly. Eta‐squared (*η* ^2^) was calculated as a measure of effect size.

Finally, correlations (Pearson) against working memory (visuospatial and verbal indices) and mathematical abilities were conducted at T2 for all the scores significantly modulated across groups and maintained over time. Even though a correlation between differential scores would have been more conclusive, the lack of WM or MATH scores at T0 impeded us from isolating the contribution of our training to the development of these abilities. However, proving their interdependency at a given time point would suggest the potential of our findings to transfer to other cognitive skills.

## RESULTS

While group assignment was conducted on the initial sample of the 144 children, we had no chance at T0 to predict how many and which children would have later dropped out. It is then important to ensure that the final sample (i.e., the three groups of 36 children each) remained matched in terms of age, cognitive, and linguistic skills at T0 to consider differences appearing at T1 and T2. On the 108 sample, the assumption of normality was not met for most of the screening variables. A non‐parametric Kruskal–Wallis indicated no significant difference among groups for age, IQ, CI, SN, and PH (all *p*s > .07). These data (see Table 2) indicated that the selected population had comparable cognitive and linguistic levels, and no confounding bias was introduced even after that 36 children dropped out from the study.

While we controlled for gender during the group assignment, the relevant drop‐out from T0 to T2 (36 children) compromised the initial gender balance across groups, but this was out of our control (see Table 3). To test quantitatively the gender bias of our final sample, we performed a 3 × 2 chi‐square test (*χ* ^2^(2, *N* = 108) = 4.22, *p* = .12) resulting not significant at *p* < .05.

**TABLE 3:** Group characteristics. The age is presented in years:months

	Assembling	Plush	Control
Sex			
Male	15	16	23
Female	21	20	13

	*M*	*SD*	*M*	*SD*	*M*	*SD*
Age	4:1	7 month	4:0	6 month	4:0	6 month

As explained in Methods, the analysis focused on the differential scores between T1, T2 relative to T0. For completeness, all raw scores at the three time‐points are reported in Table 4, whereas Table 5 reports the differential scores Delta 1 and Delta 2 for all the investigated outcomes.

**TABLE 4:** Neuropsychological evaluations at three time points. RCPM: Raven's Colored Progressive Matrices (fluid reasoning); NEPSY‐II—VS: Block Construction (visuospatial abilities); NEPSY‐II—FM: Imitating Hand Positions (fine motor abilities); I‐BST (Bus Story Test)—IS: Information Scores; I‐BST—SL: Sentence Length; I‐BST—SC: Subordinate Clauses (linguistic/narrative competence)

	T0						T1						T2					
	A		P		Ctrl		A		P		Ctrl		A		P		Ctrl	
	*M*	*SD*	*M*	*SD*	*M*	*SD*	*M*	*SD*	*M*	*SD*	*M*	*SD*	*M*	*SD*	*M*	*SD*	*M*	*SD*
RCPM	14.6	4.2	15.0	3.7	15.1	3.6	19.3	3.6	19.3	4.6	17.4	3.5	23.8	4.8	22.9	5.0	21.0	3.6
NEPSYII—VS	7.2	2.2	6.9	1.5	7.3	2.6	11.2	3.7	9.9	2.9	10.0	3.0	12.2	3.4	10.9	2.9	10.9	2.7
NEPSYII—FM	9.7	3.1	9.0	2.9	9.3	3.2	16.3	4.1	14.6	3.5	12.7	2.8	18.3	3.5	17.4	3.6	17.1	3.2
I‐BST—IS	24.0	10.8	24.0	8.8	22.1	10.3	36.4	8.1	36.1	8.0	32.8	9.8	41.5	7.2	41.1	7.9	38.8	9.3
I‐BST—SL	4.8	1.3	4.9	1.3	4.6	1.7	5.9	0.9	5.7	1.0	5.3	1.2	6.6	1.0	6.4	1.1	6.2	1.4
I‐BST—SC	1.7	1.8	1.2	1.3	1.3	1.6	3.8	2.6	3.8	2.5	2.6	2.3	4.8	2.2	4.4	2.7	3.8	2.7

**TABLE 5:** Means and standard deviations of Delta 1 and Delta 2 scores. RCPM: Raven's Colored Progressive Matrices (fluid reasoning); NEPSY‐II—VS: Block Construction (visuospatial abilities); NEPSY‐II—FM: Imitating Hand Positions (fine motor abilities); I‐BST (Bus Story Test)—IS: Information Scores; I‐BST—SL: Sentence Length; I‐BST—SC: Subordinate Clauses (linguistic/narrative competence)

	RCPM		NEPSYII—VS		NEPSYII—FM		BST—IS		BST—SL		BST—SC	
	*M*	*SD*	*M*	*SD*	*M*	*SD*	*M*	*SD*	*M*	*SD*	*M*	*SD*
Delta 1												
A	4.6	3.1	4.0	2.2	6.6	3.5	12.4	9.0	1.1	1.3	2.1	2.6
P	4.3	3.8	2.9	2.4	5.6	3.3	11.7	8.1	0.8	1.4	2.6	2.2
Ctrl	2.3	4.3	2.7	1.8	3.4	3.6	10.6	7.4	0.7	1.1	1.4	1.9
Delta 2												
A	9.1	3.9	5.0	2.5	8.6	3.6	17.5	9.2	1.8	1.2	3.1	2.5
P	7.9	4.8	3.9	2.2	8.5	3.5	16.7	7.6	1.6	1.2	3.3	2.2
Ctrl	5.9	3.6	3.6	2.9	7.8	3.3	16.7	9.6	1.6	1.5	2.5	2.4

**FIGURE 2:**
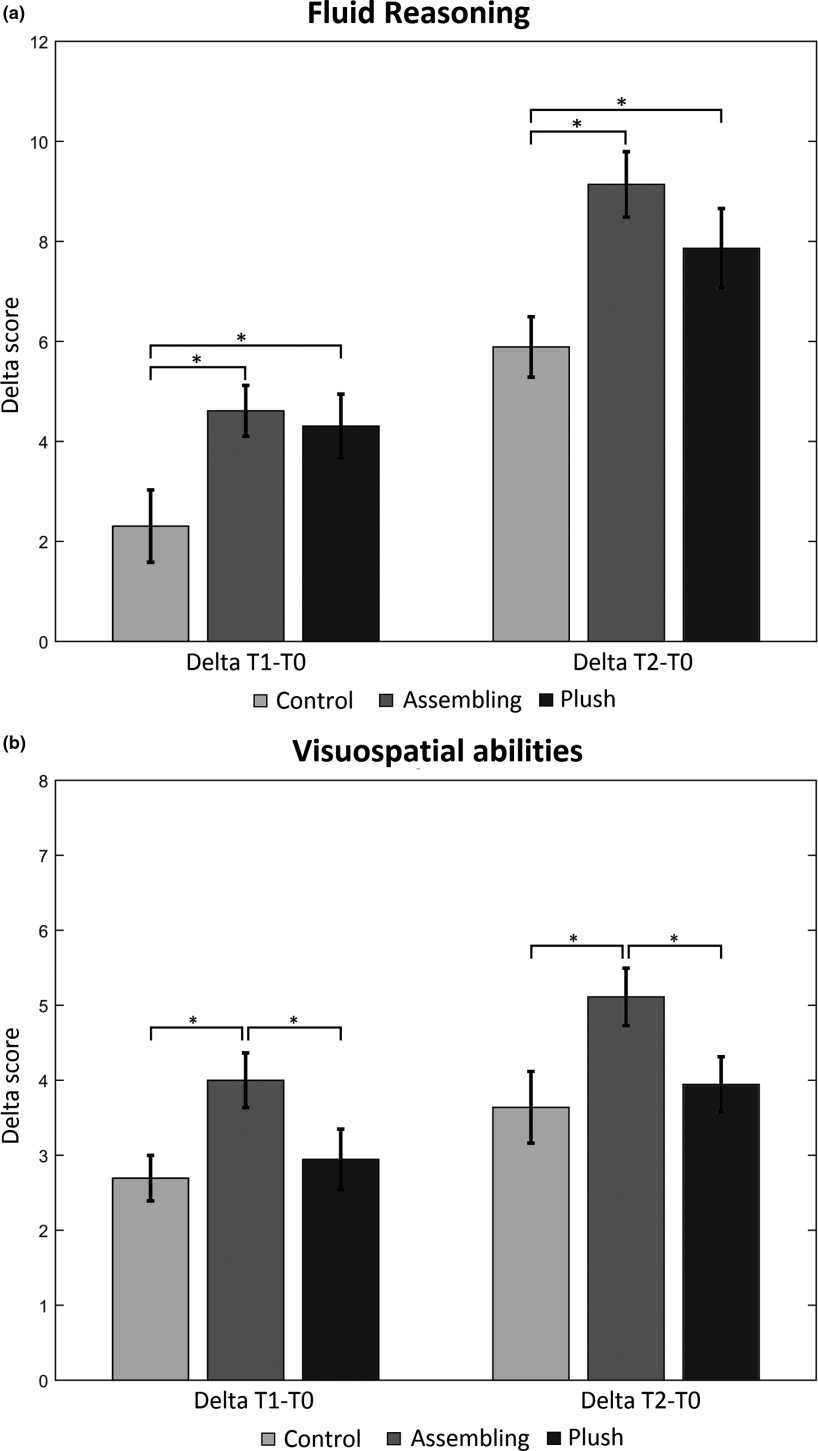
Scores for control, assembling, and plush groups at Delta 1 (T1–T0) and Delta 2 (T2–T0) for fluid reasoning (a) and visuospatial abilities (b). Bars indicate the standard error, while asterisks indicate scores significantly different between groups at post hoc analysis (*p* < .05)

The results for FR indicated a significant effect at Delta 1, *F*(2, 99) = 4.31, *p* = .02, *η* ^2^ = .075 and at Delta 2, *F*(2, 99) = 4.77, *p* = .01, *η* ^2^ = .080 (Figure 2a). Post hoc analysis (Newman–Keuls) revealed that the two experimental groups significantly differed at Delta 1 from the control group (*M* = Ctrl: 2.3, A: 4.6, P: 4.3, both comparisons *p* < .001) suggesting a specific effect of the intervention. The same pattern was obtained at Delta 2 (*M* = Ctrl: 5.9, A: 9.1, P: 7.9, both comparisons *p* < .001) showing that the effect of treatment was maintained over time.

Concerning the visuospatial scores, a significance difference was found across groups at Delta 1, *F*(2, 99) = 3.32, *p* = .04, *η* ^2^ = .055 with higher scores for the Assembling group relative to other two (*M* = Ctrl: 2.7, A: 4.0, P: 2.9, A vs. Ctrl, *p* = .02; A vs. P, *p* = .03), but no difference between Control and Plush groups (Ctrl vs. P, *p* = .61), as revealed by post hoc analysis. A similar pattern was revealed also by Delta 2 scores, with a significant group effect *F*(2, 99) = 3.67, *p* = .03, *η* ^2^ = .067 and the Assembling group maintaining a higher level of visuospatial abilities (*M* = Ctrl: 3.6, A: 5.0, P: 3.9, A vs. Ctrl, *p* = .04; A vs. P, *p* = .05) (see Figure 2b).

Regarding the motor domain, a main effect of group appeared at Delta 1, *F*(2, 99) = 7.39, *p* = .001, *η* ^2^ = .12, with post hoc reporting a specific effect for both experimental groups relative to controls (*M* = Ctrl: 3.4, A: 6.6, P: 5.6, both comparisons *p* < .001). However, the increase in motor abilities for the experimental groups was not anymore significant when examining Delta 2 scores, *F*(2, 99) = 0.35, *p* = .70, (*M* = Ctrl: 7.8, A: 8.6, P: 8.5).

Considering the linguistic/narrative domain, IS (Delta 1, *F*(2, 99) = 0.36, *p* = .70; Delta 2, *F*(2, 99) = 0.22, *p* = .81) and SL (Delta 1, *F*(2, 99) = 1.53, *p* = .22; Delta 2, *F*(2, 99) = 0.66, *p* = .52) showed no significant effect. The same happened for SC at both Delta 1 (Kruskal–Wallis: *H*(2, *N* = 108) = 5.11, *p* = .08) and Delta 2 (Kruskal–Wallis: *H*(2, *N* = 108) = 2.57, *p* = .28). Mean Delta scores are reported in Table 5. Overall, linguistic/narrative competences were poorly affected by our intervention.

In summary, we found that the intervention designed in the present study had a significant impact on FR and visuospatial abilities, whose scores remained selectively higher also at 1‐year follow‐up. In particular, both experimental groups showed a beneficial effect for FR relative to the control group. Only the Assembling group received specific training for visuospatial abilities and presented higher scores in this domain at both time points.

**FIGURE 3:**
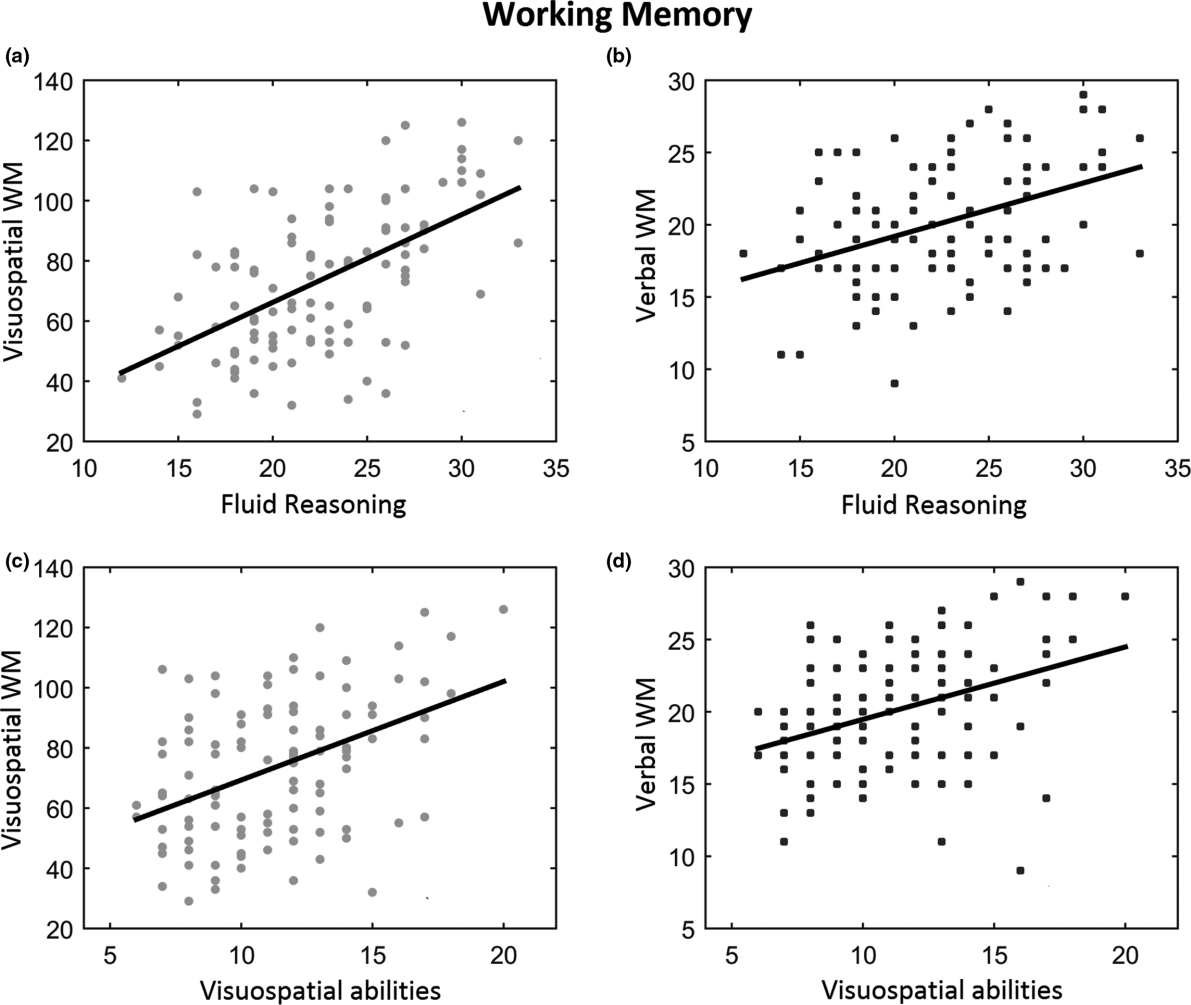
Upper part: Results of the correlation analyses between fluid reasoning and visuospatial working memory (a) scores (*r* = .57, *p* < .001), and fluid reasoning and verbal working memory (b) scores (*r* = .41, *p* < .001). Lower part: Results of the correlation analyses between visuospatial abilities and visuospatial working memory (*r* = .42, *p* < .001), left side (c) and visuospatial abilities and verbal working memory (*r* = .36, *p* < .001), right side (d). Each dot indicates a single participant. The solid black line indicates the linear fitting

Following these results, we tested whether at T2 the trained competencies could be positively correlated with working memory (visuospatial—MD, and verbal—SR) and mathematical abilities (i.e., number processing and calculation) indices. Results, reported in Figure 3, showed a significant and positive correlation between FR and MD (*r* = .57, *p* < .001) as well as with SR (*r* = .41, *p* < .001). Visuospatial abilities also positively correlated with MD, as well as with SR (*r* = .42, *p* < .001; *r* = .37, *p* < .001, respectively). Of note, in the latter case, three participants appear as outliers, as visible in Figure 3d (bottom right part of the graph). While we did not remove these participants for the sake of completeness, it is worth indicating that their deletion increases the correlation coefficient from .37 to .49, thus offering an even stronger picture of this finding.

**FIGURE 4:**
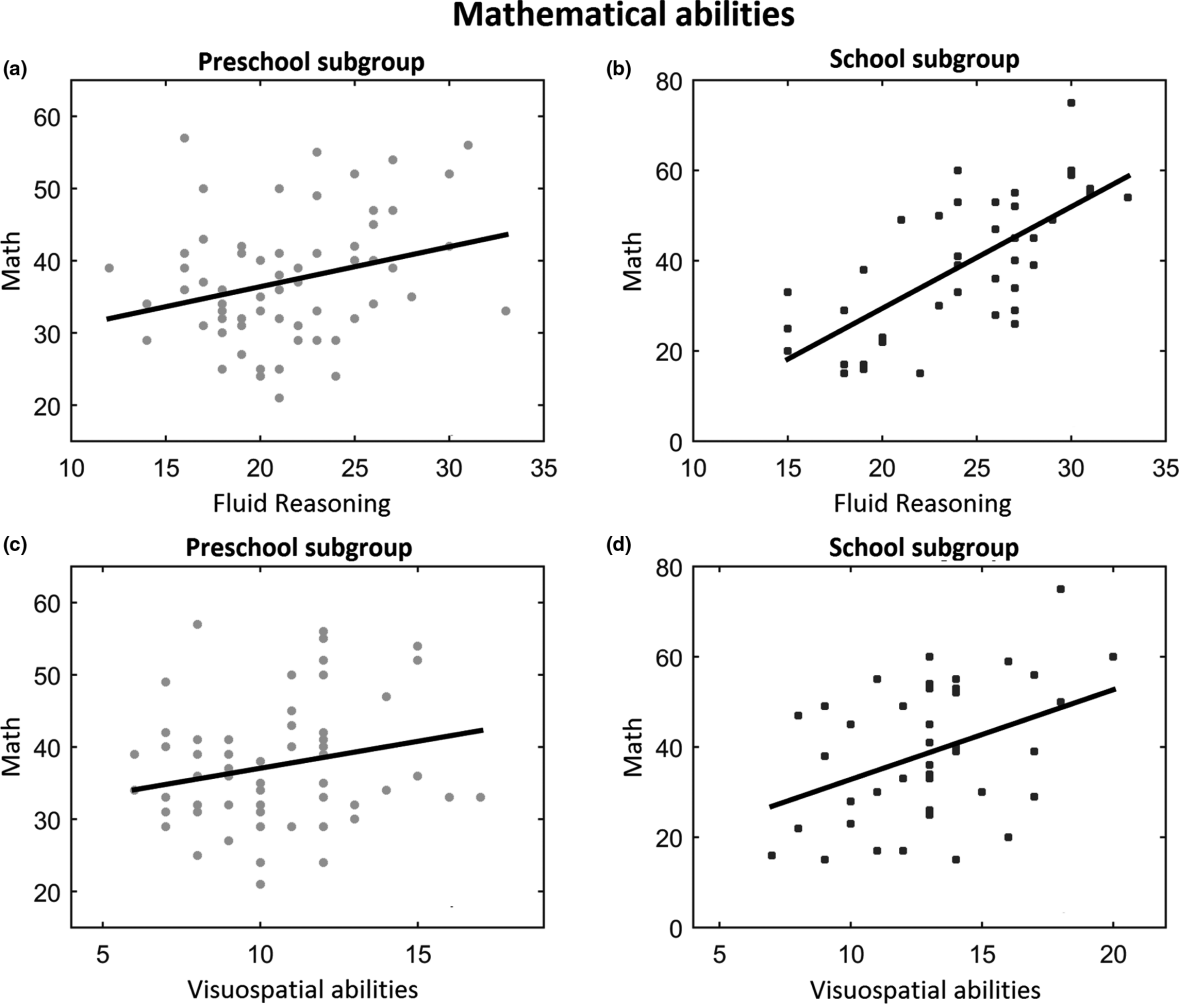
Left side: Results of the correlation analyses in preschool subgroup between fluid reasoning (a) and Math (c) scores (*r* = .28, *p* = .02), and between visuospatial abilities and Math scores (*r* = .23, *p* = .07). Right side: Correlation analyses in school subgroup between both fluid reasoning (b) (*r* = .71, *p* < .001) and visuospatial abilities (d) (*r* = .40, *p* = .01) against mathematical abilities. Each dot indicates a single participant. The solid black line indicates the linear fitting

Regarding the relation with the mathematical ability score, we split the sample into two groups according to age, as the TEDI‐MATH provides different tasks for preschool and school children. In preschool children, the correlation between FR and mathematical abilities indicated a positive correlation (*r* = .28, *p* = .02), but only a trend emerged for the correlation with visuospatial abilities (*p* = .08). In school children the analyses revealed strong, positive and significant correlations between both FR and visuospatial abilities against mathematical ones (*r* = .71, *p* < .001; *r* = .40, *p* = .007, respectively; Figure 4).

## DISCUSSION

In this study, we designed an intervention for preschool children addressing simultaneously different cognitive and motor domains yet containing features easy to transfer into everyday kindergarten practice. As the proposed intervention was centered on problem solving, narrative competencies, and visuomotor abilities, we first investigated whether its administration could enhance these domains. The results showed that FR and motor abilities were enhanced in both experimental groups (i.e., regardless of the type of toy they interacted with), while only the interaction with modular toys determined an increase in visuospatial abilities. Finally, the linguistic/narrative domain did not take advantage of the training for any group.

The second aim was to determine whether training effects were stable over time. For this purpose, all children were evaluated after 2 years since the beginning of the study. Notably, all increases in FR (for both experimental groups) and visuospatial abilities (for the Assembling group only) showed a maintenance effect, with significant effects resisting despite 1 year of non‐training. In other words, our training impacted the present cognitive abilities and set better premises for future development. The moderate effect size at T2 further reinforces the value of our findings. Looking comparatively to the two types of training, the significant and long‐lasting modulation of visuospatial abilities indicates that assembling training addresses the larger set of cognitive skills. In the next paragraphs, we will discuss each domain separately.

Fluid reasoning is considered one of the most important factors in learning, critical for a wide variety of cognitive tasks (Gray & Thompson, 2004). However, whether FR can be trained is a matter of debate. While traditionally considered a trait with a strong hereditary component (Baltes et al., 1999; Gray & Thompson, 2004) and therefore rather immune against training, recent studies succeeded in training FR (see Klauer & Phye, 2008). Our study confirms that exposing children to problem‐solving tasks enhances FR skills for at least 12/24 months. This aspect assumes fundamental importance in the debate about how durable FR training is, as increases obtained through training programs have often proved to be fleeting (Spitz, 1992). The impact of our training on FR was durable, not vanishing shortly after the end of the training, potentially setting better premises for the development of other cognitive abilities and later professional and educational success (Deary et al., 2007; Neisser et al., 1996).

While a detailed comparison of our intervention relative to the procedures previously reported in the literature is virtually impossible, two peculiar aspects may have contributed to its success in modulating FR. The first is represented by the social context in which the training took place, contrary to computerized training programs to be performed individually (Bergman Nutley et al., 2011; Mackey et al., 2011). The social context may have driven imitative behaviors and boosted motivation to participate in the activities. The relational processes that occur when young children engage with others constitute a platform for advancing children's cognitive abilities. The second aspect is represented by the play‐like setting, highly distant from the laboratory‐ or class‐like environments, instantiated by toys and their enactment into the shared, narrated story.

Our training also enhanced fine‐motor abilities. A specific effect was expected only for the Assembling group, whose children spent the time in activities (e.g., building a toy) that required fine motor competencies. After the training, an improvement was found in both experimental groups, but this effect vanished at T2. The unspecificity and fleetingness of these findings might be linked to an insufficient time of exposition or inadequate sensitivity of the test used for the evaluation. Indeed, 32 play sessions might not suffice to make an increase in fine motor abilities emerge for the group exposed to modular toys. While the delivered amount of training seemed initially relevant, the young age of the experimental groups, together with the longer maturation time required by fine‐motor skills relative to the gross‐motor ones (see Gasser et al., 2010), may have blurred the expected outcome. A complementary explanation concerns the test adopted for the motor evaluation. The Imitating Hand Position subtest (NEPSY‐II) is designed to assess the ability to imitate static hand and finger configurations. Thus, it is probably more sensitive to postural imitation skills than abilities underlying fine and sequential movements. Despite this globally negative result, indexing fine motor skills in preschool children is fundamental given the relevance in driving later development. For this reason, future studies should consider using longer or more intensive training and the adoption of neuropsychological or neuro‐motor tests more sensitive to subtle increases of motor functioning (see Movement Assessment Battery for Children—Second Edition, Henderson et al., 2007).

A clear difference between experimental groups emerged after the training in the visuospatial domain. Indeed, to construct their toys, children of the Assembling group, but not children of the Plush group, had to follow visual instructions, commuting 2D visual images into 3D toys. This activity selectively involves the individual's capacity to manipulate and transform visual information to obtain a final goal. The specificity of the increase for the Assembling group supports the idea of the malleability and upgradability of visuospatial skills after specific training. Similar conclusions were reached by Casey et al. (2008) investigating the use of block‐building interventions to develop spatial‐reasoning skills in children of the same age as in this study.

Although the retelling represented one important element of our intervention, no significant impact was found in the linguistic/narrative domain. Indeed, I‐BST scores increased along observation times but with no modulation across groups (see Table 1; Supporting Information). This finding could be due to the low dosage of narrative training administered to children. In other words, 32 sessions in a year may not have been capable of super‐adding a meaningful enhancement to the physiological development of linguistic abilities, which are daily trained in educational and social environments.

In conclusion, our training during preschool years sustains the emergence of FR and visuospatial abilities and their maintenance over time. Using a correlative approach, we highlighted positive correlations of these scores against mathematical skills and working memory.

The link between spatial abilities and mathematics is well established (e.g., Dehaene et al., 1999), even if different hierarchies have been proposed. On one side, spatial reasoning could overlap and serve as a premise for mathematical reasoning skills (Tosto et al., 2014). On the other, spatial abilities and mathematics would be based on shared underlying processes (see Hubbard et al., 2005). A large series of previous studies revealed that children and adults who perform better on spatial tasks also perform better on tests of mathematical ability (Cheng & Mix, 2014; Holmes et al., 2008; Worrell et al., 2020). Focusing on young children, Mix et al. (2016) enrolled 854 children (5–13 years old), revealing that different spatial abilities are associated with better mathematical performance in a time‐dependent manner during early and late childhood. Indeed, while mental rotation is the best predictor of mathematical performance in kindergarten, visuospatial working memory is the best in sixth grade (i.e., 11–12 years old). However, the link between spatial abilities and mathematics is robust throughout the entire school age, from kindergarten to 12th grade (i.e., 17–18 years old), with performance in mental rotation tasks serving as the best predictor of mathematical skills (Lachance & Mazzocco, 2006; Thompson et al., 2013). The strength of such a link made researchers explore whether interventions on visuospatial abilities transfer to mathematical skills. Wolfgang et al. (2001) found that preschool children who engage in more block play perform better in school math, even if this effect appears only during high school. Similar findings were also reported by Mix and Cheng (2012).

A tight relation also exists between FR and mathematics. This is not surprising (McGrew & Hessler, 1995; Taub et al., 2008), considering that FR and math problems engage common underlying cognitive processes and sustain the ability to account for multiple relations among the components of a problem (Halford et al., 1998; Miller Singley & Bunge, 2014).

The correlative analyses conducted at T2 indicated that our training's major outcomes were significantly associated with mathematics and working memory. Its association with visuospatial abilities has been witnessed by previous behavioral and neuroimaging reports (Kyttälä & Lehto, 2008; Levin et al., 2005; Shah & Miyake, 1996), and analog parallelisms have been shown between working memory and FR (see for a review Yuan et al., 2006). The correlative analyses aimed to confirm the existence of the abovementioned relation in our sample. As this was the case, we can hypothesize to have induced indirect yet beneficial effects on these domains.

## CONCLUSIONS AND LIMITATIONS

We designed an intervention capable of enhancing emerging cognitive functions like FR and visuospatial abilities, further sustaining their maintenance over time. Moreover, the correlations with visuospatial working memory and mathematical skills suggest a secondary effect on other cognitive domains. The proposed intervention is relatively easy to be conducted with preschool children; it stresses their natural cooperative attitude, is embedded into a play‐like context promoting motivation and compliance, and, more importantly, stimulates different cognitive domains simultaneously. Thus, even in the daily preschool practice, it seems well suited to accompany young children toward the potentiation of emerging skills and the acquisition of new ones fundamental for their future learning and discoveries.

A strength of our study was that sampling was not limited to a pre‐post design but rather envisioned a longitudinal evaluation carried out at three times (T0, T1, and T2) on all children. However, the results should also be considered against the limitations of the study. The first limitation of our study regards the poor sensitivity of the Imitating hand position subtest (IH) in measuring fine motor abilities. The choice of each test was guided by the need to keep the overall testing duration reasonable. Classical neuro‐motor evaluations generally require a long time to be administered. However, indexing fine motor skills in preschool children is fundamental given their relevance in later development. For this reason, future studies should consider adopting neuropsychological or neurological tests more sensitive to fine‐motor abilities (see Movement Assessment Battery for Children—Second Edition, Henderson et al., 2007).

The second limitation concerns the lack of mathematical and working memory assessment at T0 and T1, impeding us from investigating whether our training indirectly enhanced these functions. Beyond the temporal constraint mentioned above, it is worth noting that the Tedi‐Math is indicated for children of 4 years or older. As half of our initial sample was younger than 4, Tedi‐Math would have provided disputable results at T0 or T1. Future studies could face this point by selecting different evaluation tests.

One could wonder whether a larger sample size would have returned stronger results. While we cannot discard this point, most statistical comparisons indicated significant effects and at least moderate effect sizes. Negative findings, on the contrary, appear not related to an insufficient sample size but rather to biases in the experimental design. A valid argument instead would be that all children have been recruited in the same town. Larger recruitment, possibly including children from different regions, could grant more reliable and generalizable results. No prejudice, however, stands against the applicability of our findings to other regions, indicating a good generalizability to different geographic contexts. On the contrary, a larger sample would likely have participants with different socio‐demographic backgrounds (information not collected in our study), highlighting its potentially modulatory role on training effectiveness. In summary, we cannot neglect that we recruited a good sample size yet narrow in several factors impacting the children's cognitive development. A much larger sampling exploring multiple backgrounds, different IQ levels (e.g., below‐average, average, and above‐average), and socio‐demographic conditions would be fundamental to make our findings generalizable for preschoolers.

As evaluation had to be applied to children since 3 years old, a non‐verbal IQ test was identified. Besides, it had to be different from the Raven test that would have served later in the evaluation. However, it is well‐documented how the Leiter‐R test overestimates IQ scores relative to other standard tests (Grondhuis & Mulick, 2013), possibly due to the non‐verbal nature of the requested items. This aspect has to be carefully accounted for in the data analysis and their interpretation against reference values.

Finally, the two interventions allowed us to isolate effects specifically driven by modular toys. Still, children could have been attracted differently by the interaction with modular or plush toys. The whole experimental design was kept identical for the two groups, including the characters of the toys, just to minimize this potentially confounding effect. For future applications, it would be recommended to collect data concerning children's engagement into the different arms of the intervention, thus verifying their substantial homogeneity.

## CONFLICT OF INTEREST

The authors declare no competing interests.

## AUTHOR CONTRIBUTION

V.G., M.F.‐D., and G.R. designed the experiment. V.G., M.C.B., C.M., and P.P. performed data acquisition and analyses. V.G., M.F.‐D., and G.R. interpreted the results and wrote the paper. All authors have contributed to, seen, and approved the manuscript.

## Supporting information

Supplementary Material
