# Supplementary material for: Supporting preschoolers’ cognitive development: Short‐ and mid‐term effects of fluid reasoning, visuospatial, and motor training
Source: Child Dev. 2021 Aug 20;93(1):134–49. doi: 10.1111/cdev.13642 (PMC9291496; doi:10.1111/cdev.13642)

**Lucio the seahorse**

**Characters:**

Characters played by the experimenter:

- Lucio
- Lino
- Orca

Characters played by the children:

- Marine animals (i.e. Lucio classmates)

Once upon a time, in Corals City, in the depths of the ocean, the seahorse Lucio attended school with his friends. He was very generous and nice with all his schoolmates, and he was always ready to help his friends in trouble.

*Lucio: “do you need help? Are you hurt little fish?”*

During the break time, Lucio and his friends were used to eat their snacks while playing hide-and-seek over the reef. But every day the spiteful and overbearing fish Lino was used to show up and play tricks on them. Lino teased his younger companions, demanded to eat their snacks, imposed the game to play and while swimming he used fins to push his mates in the middle of the coral.

Question Time

SEMANTIC QUESTION:

DO YOU KNOW WHAT FINS ARE (children are invited to respond individually)?

*[Fins are the thin parts that stick out from the body of a water animal and especially a fish and are used in moving or guiding the body through the water]*

Lino: “*No, you can’t play hide-and-seek, I want to decide the game.” “Now I will steal your delicious snack!”* continued Lino waving his tail.

One day, while Lino was bothering his classmates, a giant animal arrived from the depth of the ocean, she was a big orca…

*[The experimenter enacts the arrival of the big and threatening orca using his character]*

Question Time

HYPOTETICAL QUESTION:

AND WHAT HAPPENED NEXT?

The big orca was famous for being greedy of little fishes and for this reason all of them were scared by her.

As soon as Lucio, Lino and their friends saw her, they escaped in all directions to hide.

Lucio: “*Friends, I’ve got an idea! I’ll let her follow me so you can run away and save yourselves!”*

*“Orca, orca I’m here!! Come and get me, if you dare!”* shouted the seahorse.

The big orca replied to him: “*I’ll be there in a blink of an eye, and I’ll eat you in one bite! Ah ah ah!”*

*[The experimenter enacts the orca chasing Lucio]*

All the fishes swam as fast as possible to save themselves, hiding behind the algae and the corals. Lucio, on the other hand, continued to be chased for days and days.

The little seahorse was very tired and he started to lose his strengths. His friends wondered what they could do to help Lucio. “*Lucio has been chased by the orca since too long and she’s about to reach him. What can we do to help Lucio?”* said the blue fish.

*“We all can do something to help him!”* answered Lino.

Question Time

RESOLUTION OF UNEXPECTED EVENTS QUESTION:

WHAT CAN THEY DO TO HELP LUCIO? (children are invited to respond individually)

Lino said “*Look! There’s a lot of algae there, what can we build to capture the orca? We can gather the algae and tie them together with our fins, so to build a net to trap the orca.”*

*“*[*Is*](https://it.pons.com/traduzione/inglese-italiano/Is) [*that*](https://it.pons.com/traduzione/inglese-italiano/that) [*a*](https://it.pons.com/traduzione/inglese-italiano/a) [*good*](https://it.pons.com/traduzione/inglese-italiano/good) [*idea*](https://it.pons.com/traduzione/inglese-italiano/idea)*?*” [asked](https://it.pons.com/traduzione/inglese-italiano/said) [Lino](https://it.pons.com/traduzione/inglese-italiano/Lino).

**LOGICAL TASK – repeated pattern**

*We have to help Lucio friends building the algae net. Let’s place the algae in the correct order.*

*First we put the red algae, then the green one and then the purple one. Now we put the red algae again, and then? Try to complete the sequence with the remaining cards.*

**

*“Well done! Each of us contributed to achieve the final goal!*” said Lino, leaving all the fish astounded.

Question Time

ATTRIBUTION OF STATE QUESTION:

DO YOU THINK THAT LUCIO KNOWS THAT HIS FRIENDS HAVE BUILT A NET?

WHY? (children are invited to respond individually)

No! Lucio didn’t know that Lino and friends had built a net, because he was busy distracting the orca and he had not seen them. The little seahorse thought he would have had to run away still for a long time because he did not know that his friends had a plan to save him.

*“Now let’s call Lucio and tell him our plan”* Said one of the little fishes.

*“Lucio, Lucio!”* called Lino. *“Lucio can’t hear me, let’s call him all together!”* said the little yellow fish.

*“Lucio, Lucio!”* the fishes screamed.

“*Hi friends!”* replied Lucius

*“We have built a net with algae, and we have a plan to trap the orca. As you are very little, you can get closer to the net and swim through its holes. The orca instead will not get through and will be trapped”* said Lino.

All the fishes hold the net, and Lucio swam safely through the net while the big orca remained imprisoned. Lucio was safe and felt grateful to Lino, the spiteful fish. The little seahorse thanked everyone saying “*thank you friends and especially to you, Lino*!”

*“Thanks to you Lucio, you saved us from the orca. I realized that I do not have to be spiteful and overbearing, and that I can use my fins to help others.”*

All fishes were happy with Lino’s change and Lucio’s comeback, so they went back to school and started playing together hide-and-seek.

Children are invited to retell the story individually

**MORAL OF THE STORY**

**What has this story taught us?**

*It is important to help each other and cooperate, especially when a friend of ours is in trouble.*

**LOGICAL TASK CARDS**

The experimenter places the first 4 cards in the correct sequence:

**red algae – green algae – purple algae – red algae**

Then he puts the other cards randomly on the table and asks the children to complete the sequence in the correct order.


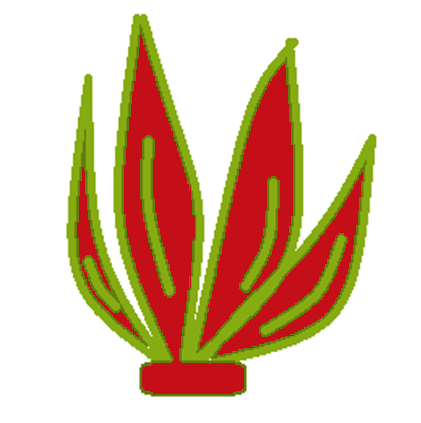


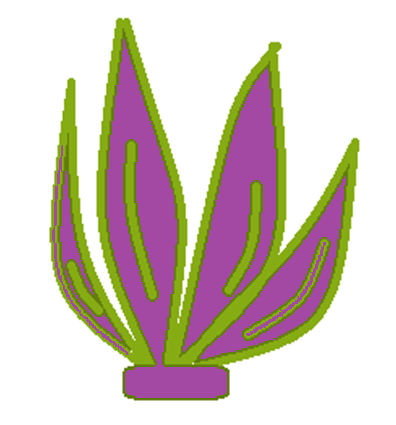


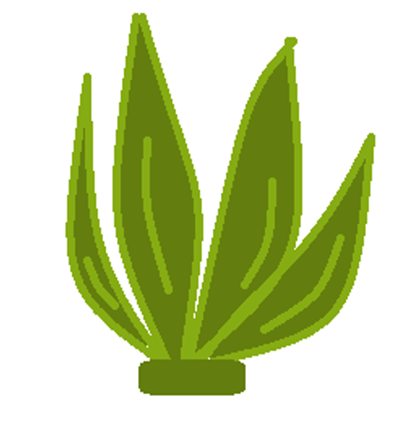


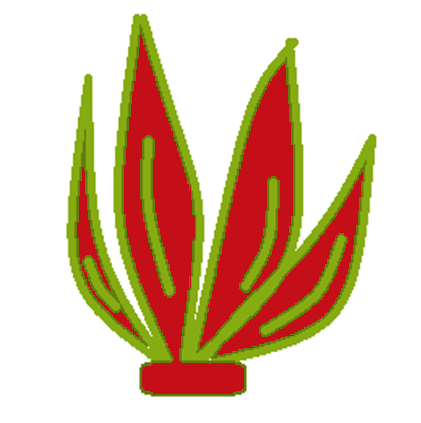

Supplement: Supplementary file 1 — Supplementary Material [file CDEV-93-134-s001.docx]
